# Integrating AIPSS‐MF and molecular predictors: A comparative analysis of prognostic models for myelofibrosis

**DOI:** 10.1002/hem3.60

**Published:** 2024-03-20

**Authors:** Adrián Mosquera‐Orgueira, Eduardo Arellano‐Rodrigo, Marta Garrote, Iván Martín, Manuel Pérez‐Encinas, María‐Teresa Gómez‐Casares, Alberto Hernández‐Sánchez, Francisca Ferrer‐Marín, Elvira Mora, Patricia Velez, Rosa Ayala, Anna Angona, Natalia de las Heras, Elena Magro, Carlos Pérez‐Míguez, Davide Crucitti, María‐Isabel Mata‐Vázquez, María‐Laura Fox, Sonia González de Villambrosía, María‐José Ramírez, Ana García, Valentín García‐Gutiérrez, Amparo Cáceres, María‐Antonia Durán, María‐Alicia Senín, José‐María Raya, José A. González, Beatriz Cuevas, Blanca Xicoy, Jyoti Nangalia, Jesús M. Hernández‐Rivas, Beatriz Bellosillo, Alberto Álvarez‐Larrán, Juan C. Hernández‐Boluda, Spanish MPN Group (GEMFIN)

**Affiliations:** ^1^ Santiago de Compostela Hospital Clínico Universitario Valencia Spain; ^2^ Hospital Clínic, Institut d'Investigacions Biomèdiques August Pi i Sunyer Barcelona Spain; ^3^ Hospital Clínico Universitario‐INCLIVA, University of Valencia Valencia Spain; ^4^ Hospital Dr Negrín, Las Palmas de Gran Canaria Las Palmas de Gran Canaria Spain; ^5^ Hospital Clínico Salamanca Spain; ^6^ Hospital Morales Meseguer Universidad Católica San Antonio de Murcia, Centro de Investigación Biomédica en Red de Enfermedades Raras Murcia Spain; ^7^ Hospital Universitario La Fe Valencia Spain; ^8^ Hospital del Mar Barcelona Spain; ^9^ Hospital Universitario 12 de Octubre, I+12, Complutense University, Centro de Investigación Biomédica en Red de Oncología Madrid Spain; ^10^ Hospital Josep Trueta Institut Catalá d'Oncologia Girona Spain; ^11^ Hospital Universitario de León León Spain; ^12^ Hospital Príncipe de Asturias, Alcalá de Henares Alcalá de Henares Spain; ^13^ Hospital Costa del Sol Marbella Spain; ^14^ Vall d'Hebron Institute of Oncology (VHIO) Vall d'Hebron Hospital Universitari, Vall d'Hebron Barcelona Hospital Campus Barcelona Spain; ^15^ Hospital Marqués de Valdecilla Santander Spain; ^16^ Hospital General, Jerez de la Frontera Jerez de la Frontera Spain; ^17^ Hospital Clínico Universitario Valladolid Spain; ^18^ Hospital Ramón y Cajal Madrid Spain; ^19^ Hospital Arnau de Vilanova Valencia Spain; ^20^ Hospital Son Espases Mallorca Spain; ^21^ Institut Catalá d'Oncologia L'Hospitalet de Llobregat Spain; ^22^ Hospital Universitario de Canarias Tenerife Spain; ^23^ Hospital Virgen del Puerto Plasencia Spain; ^24^ Hospital Universitario de Burgos Burgos Spain; ^25^ Hospital Germans Trias i Pujol, Institut Català d'Oncologia, Josep Carreras Leukemia Research Institute Universitat Autònoma de Barcelona Badalona Spain; ^26^ Wellcome Sanger Institute Hinxton UK

Myelofibrosis (MF) is a chronic myeloproliferative neoplasm that can manifest as a primary condition (primary myelofibrosis [PMF]) or after progression from a polycythemia vera or essential thrombocythemia (secondary myelofibrosis [SMF]). The aberrant activation of the JAK‐STAT pathway is central to MF pathogenesis which is caused by driver mutations in *JAK2*, *CALR*, and *MPL* genes. These mutations, along with additional somatic variants that mainly impact epigenetic modifiers or spliceosome components, shape the clinical features of the disease.^1^

Although the median overall survival (OS) is around 6 years, the clinical course of MF is heterogeneous. The only curative strategy, allogeneic hematopoietic cell transplantation, carries a significant risk of early mortality.^2^ It is therefore critical to accurately assess transplantation risk and estimate survival with medical therapies to determine the most appropriate treatment approach for each individual.^3^

Several prognostic models are available to categorize patients into risk groups.^4^, ^5^, ^6^, ^7^, ^8^, ^9^, ^10^ Despite their utility, these models have limitations, such as exclusive applicability to specific MF subtypes, the need for karyotypic analysis, which may be challenging due to insufficient bone marrow aspiration, or reliance on Next Generation Sequencing (NGS) techniques that may not be widely accessible.

To address these limitations, we recently conducted a study involving 1617 MF patients from 60 Spanish institutions. In this study, we employed a machine learning (ML) method to develop the AIPSS‐MF (Artificial Intelligence Prognostic Scoring System for Myelofibrosis; available at https://geneticsoncohematology.com/MF/).^11^, ^12^ This model, which relies on eight clinical variables (age, sex, hemoglobin, leukocytes, platelets, peripheral blasts, constitutional symptoms, and leukoerythroblastosis), evaluated at MF diagnosis, demonstrated a robust capability to predict OS and leukemia‐free survival (LFS). Notably, its predictive accuracy surpassed that of established prognostic models like the IPSS for PMF patients and the MYSEC‐PM for SMF patients. One of the key advantages of the AIPSS‐MF relies on its ability to provide personalized risk estimates for each patient. Furthermore, the model is based on clinical rather than genomic data, making it suitable for implementation in most healthcare settings.

However, the potential improvement of our ML model's prognostic accuracy by incorporating molecular data on additional somatic mutations could not be adequately evaluated because a significant proportion of patients did not have this information available at that time. To address this gap, we have conducted a new study including 581 MF patients from the GEMFIN database who had available NGS annotation.

DNA samples were isolated from peripheral blood, mostly within the first year of MF diagnosis (58%). Targeted NGS sequencing was performed locally, although 450 (77%) of cases were analyzed at 9 referral centers. Despite evaluating up to 56 genes, only 20 were consistently analyzed across the different NGS panels (missing rate <10%, Supporting Information S1: Table 1). We considered only pathogenic or likely pathogenic variants with a variant allele frequency (VAF) ≥ 1%. Characteristics and outcomes of the patient cohort are shown in Supporting Information S1: Table 2.

We employed random survival forest models to predict OS and LFS, focusing on the 20 genes with data availability exceeding 90%.^13^ First, three different ML models were evaluated based solely on NGS results without taking into account clinical data. The initial model considered the mere presence or absence of mutations in each gene. Subsequently, the second model was constructed based on the cumulative number of mutations per gene. The third model focused on the VAF of each mutation, aggregating VAFs when multiple mutations affected a single gene for a comprehensive representation. We aimed to fit these models across the entire cohort to optimize prediction precision. The primary metric for accuracy assessment was the out‐of‐bag (cross‐validated) Harrel's c‐index. Notably, we employed an iterative elimination of less impactful variables to reduce dimensionality. The ML‐derived molecular predictors were compared with AIPSS‐MF, IPSS, and MIPSS70 scores using bootstrapped c‐indexes, implementing 500 bootstrap iterations. Classification of myelodepletive versus myeloproliferative MF was based on criteria by Coltro et al.^14^

For OS prediction, the ML model considering VAF proved superior to those based solely on the presence/absence of mutations or the total mutation count per gene (Supporting Information S1: Table 3). The model's accuracy was slightly augmented by incorporating data on *CALR* mutations and *U2AF1* Q157 mutation. Subsequent variable reduction resulted in a refined model that comprised the VAF of 16 genes, achieving a c‐index of 0.653, which we named the *NGS model for overall survival*. This streamlined model underscored the significance of specific genes, such as *TP53*, *SRSF2*, and *EZH2* (Figure 1A). In parallel, the primary model for LFS prediction was based on the VAF of 20 genes (c‐index, 0.702; Supporting Information S1: Table 3) and showed slight improvement when mutational data of the *CALR* gene or *U2AF1* Q157 mutation was incorporated. Unlike the OS model, the LFS model's accuracy declined upon attempting to reduce the number of variables, leading us to retain the original model, which we named the *NGS model for leukemia‐free survival*. The genes with the greatest impact in this model were *TP53*, *EZH2*, *IDH1*, *U2AF1*, *RUNX1*, *CBL*, *SRSF2*, and *IDH2* (Figure 1B). Importantly, our analysis showed consistent results when considering the time lapse between NGS analysis and MF diagnosis, with c‐indexes of 0.691 and 0.706 for OS and LFS predictions, respectively.

**Figure 1:**
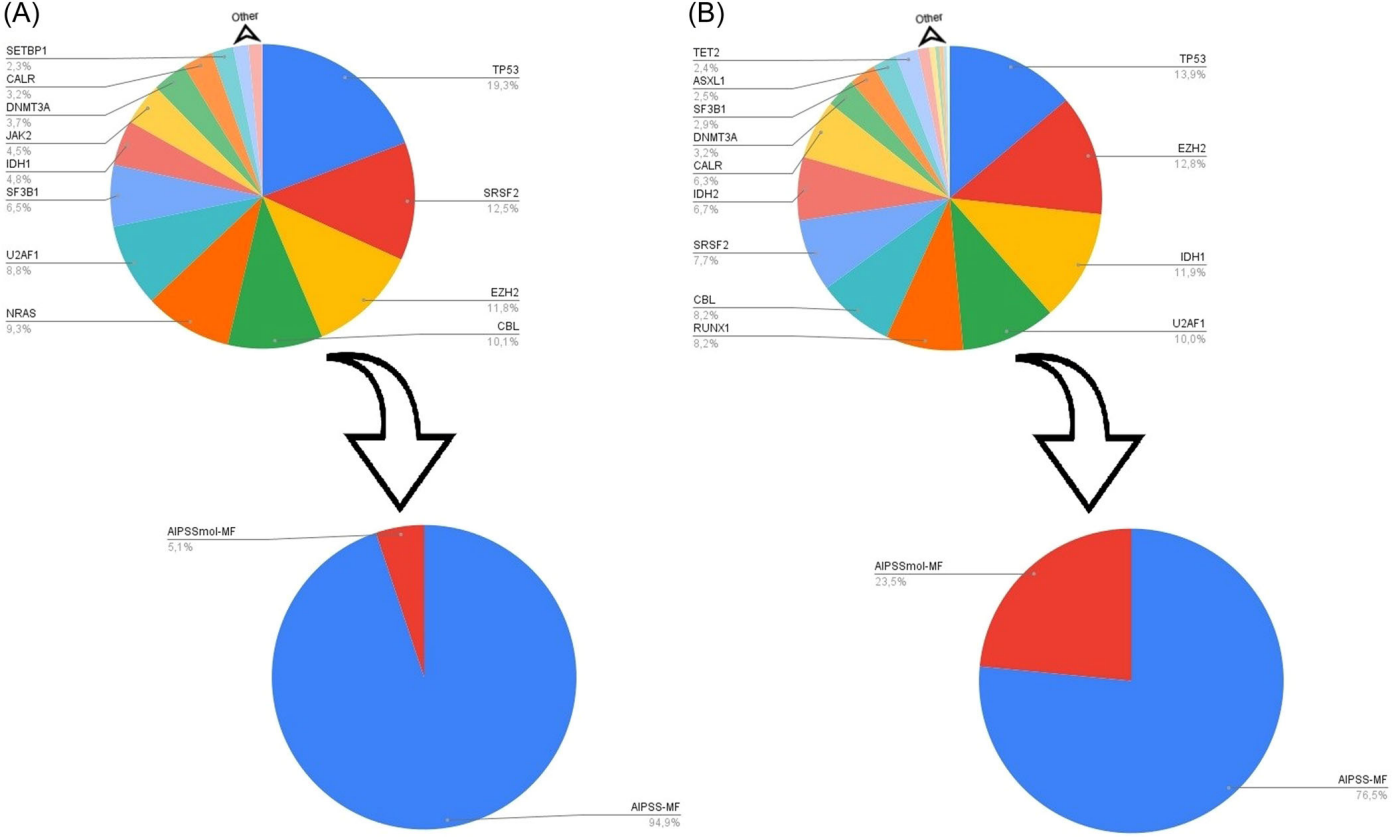
(A) Pie charts representing the relative importance of mutations in the 16 genes that contributed most to the overall survival predictor (*NGS model for overall survival*), and integration with the AIPSS‐MF clinical risk score (AIPSSmol‐MF^Surv^). Larger segments correspond to higher relative importance of the variable in the model. Genes included in this model were *ASXL1*, *CALR*, *CBL*, *DNMT3A*, *EZH2, IDH1, JAK2, KRAS, MPL, NRAS, SETBP1, SF3B1, SRSF2, TP53, U2AF1*, and *ZRSR2*. (B) Pie charts representing the relative importance of the mutations that contributed most to the leukemia‐free survival predictor (*NGS model for leukemia‐free survival*) and integration with the AIPSS‐MF clinical risk score (AIPSSmol‐MF^Leuk^). Genes included in this model were *ASXL1, CALR, CBL, DNMT3A, EZH2, IDH1, IDH2, JAK2, KIT, KRAS, MPL, NRAS, RUNX1, SETBP1, SF3B1, SRSF2, TET2, TP53, U2AF1*, and *ZRSR2*, along with *CALR* mutation type I versus II and *U2AF1* Q157 mutation type.

When comparing the performance of the AIPSS‐MF score with the *NGS model for overall survival* across the entire cohort, AIPSS‐MF demonstrated superior accuracy (bootstrapped c‐indexes of 0.812 vs. 0.649). Combining both predictors (hereafter referred to as the AIPSSmol‐MF^Surv^ model) resulted in a modest increase in the c‐index to 0.816 (Figure 1A). Results remained consistent after excluding the original training set of the AIPSS‐MF score (177 patients), (Supporting Information S1: Table 4). Compared to IPSS and MIPSS70 in a subset of adequately annotated patients (*N* = 511), AIPSS‐MF yielded the highest c‐index (0.814 vs. 0.724 for IPSS and 0.654 for MIPSS70). Incorporating the ML molecular predictor marginally enhanced AIPSS‐MF's accuracy (c‐index, 0.817) but notably boosted IPSS and MIPSS70 scores (c‐index, 0.747 and 0.696). These findings were consistent across different patient groups, including those under 70 years of age or diagnosed with PMF, regardless of transplant status (Supporting Information S1: Table 5). While all scores displayed suboptimal performance in myelodepletive MF compared to myeloproliferative MF, AIPSS‐MF remained at the top. Furthermore, the addition of the ML molecular predictor to AIPSS‐MF did not improve accuracy in myelodepletive MF patients.

We then integrated the AIPSS‐MF score with the *NGS model for leukemia‐free survival* creating the AIPSSmol‐MF^Leuk^. This model showed moderate improvement over the AIPSS‐MF score alone (AIPSS‐MF c‐index, 0.756; AIPSSmol‐MF^Leuk^ c‐index, 0.791; Figure 1B). Both AIPSS‐MF and AIPSSmol‐MF^Leuk^ performed better than IPSS and MIPSS70, particularly in patients ≤70 years and in those with myelodepletive MF. Of note, the *NGS model for leukemia‐free survival* demonstrated superior accuracy for LFS prediction in PMF than in SMF (Supporting Information S1: Table 6).

We subsequently conducted a comparative analysis between the newly generated scores and the 5, 10, and 20‐year survival predictions using Grinfeld et al.'s method (*blood.predict.nhs.uk*).^10^ Predictions were generated in the absence of cytogenetic annotation and using only the available genetic data (Supporting Information S1: Table 7). Our findings revealed that, when considering the clinical AIPSS‐MF score in isolation, it slightly outperformed the Grinfeld score in predicting OS and LFS. Notably, the superiority of the AIPSS‐MF score in forecasting LFS was greater when integrating NGS data.

In the present study, we leveraged a large database comprising 581 MF patients from academic and non‐academic institutions in Spain, which provides a realistic reflection of real‐world MF outcomes in a healthcare system with universal coverage. However, several methodological limitations require consideration. First, the variety of NGS panels used constrained our analysis to 20 genes, potentially overlooking other important genetic factors. The absence of a centralized mutation review increases the risk of interpretational disparities. Another limitation is the absence of cytogenetic information in our model, due to the significant proportion of cases without an informative karyotype. Finally, although we mitigated the absence of an external dataset by cross‐validating our findings, the intrinsic limitations of internal validation loom.

Our research has set a new advance in MF prognostics by revising clinical‐genomic models tailored to individualized risk assessments. Our models, which take advantage of the power of mutation VAFs, have superior performance to traditional methods that focus merely on the presence or absence of mutations or their cumulative count.

Our results reinforce the significant prognostic role of TP53, spliceosome, and RAS pathway mutations, while reducing the relevance of *ASXL1* mutations, aligning with the latest findings in the field.^15^, ^16^, ^17^ Notably, the AIPSS‐MF model has consistently outperformed established scores like IPSS and MIPSS70 in predicting OS. Furthermore, the integration of molecular data into this model has yielded modest yet significant improvements, particularly in predicting LFS.

Our findings advocate for the inclusion of NGS data in prognostic assessments, where available, to refine LFS predictions. This integration holds significant potential for clinical decision‐making, especially in determining the ideal timing for transplantation in younger MF patients. To bridge the gap between research and clinical practice, we have developed an accessible online calculator (Figure 2), (available at https://molecular-aipss-mf.prod.gemfin-env.gemfin.click/). This tool represents a step toward personalized medicine, offering a more accurate and individualized approach to MF management.

**Figure 2:**
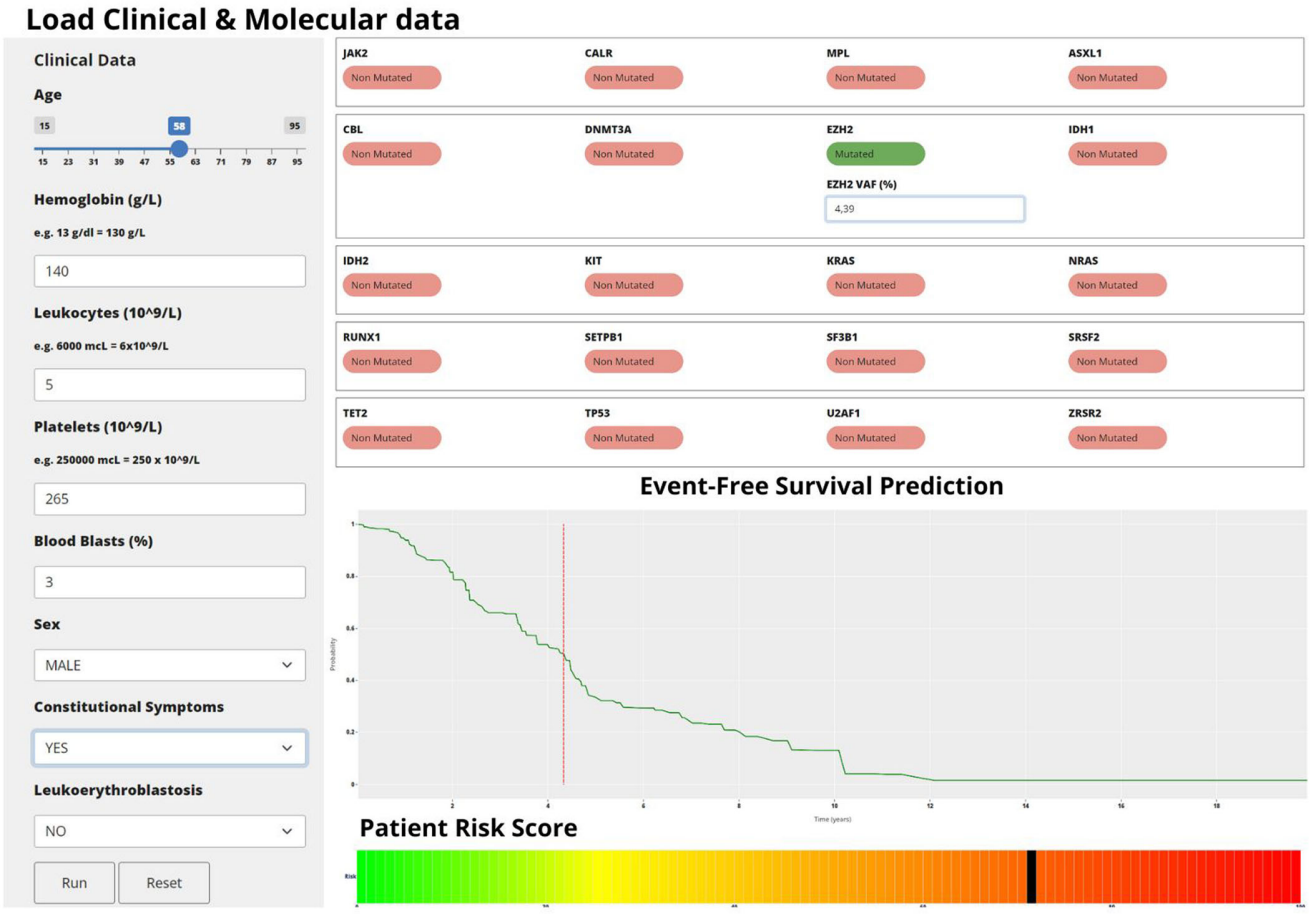
Illustration of a web‐based calculator for computing the scores discussed in this study.

In summary, our study not only contributes to the existing body of knowledge on MF prognostication but also paves the way for more tailored and effective treatment strategies, enhancing the quality of care for patients with this complex condition.

## AUTHOR CONTRIBUTIONS

Juan C. Hernández‐Boluda prepared the GEMFIN database. Adrián Mosquera‐Orgueira performed the machine learning analysis. Jyoti Nangalia calculated risk scores according to Grinfeld et al.'s method. Adrián Mosquera‐Orgueira, Manuel Pérez‐Encinas, and Juan C. Hernández‐Boluda wrote the paper. All coauthors critically evaluated the manuscript, made substantial recommendations, and approved the submission of this manuscript.

## CONFLICT OF INTEREST STATEMENT

The authors declare no conflict of interest.

## FUNDING

The Spanish Registry of Myelofibrosis was initially sponsored by a grant from Novartis Pharmaceuticals, Inc. The study was approved by the scientific board of GEMFIN. Funding for a fraction of the NGS analysis was provided by "Proyectos de investigación del SACYL", Spain, GRS 2509/A/22.

## Supporting information

Supporting information.

## Data Availability

The data that support the findings of this study are available on request from the corresponding author. The data are not publicly available due to privacy or ethical restrictions. The data supporting the findings of this study are not publicly available due to privacy or ethical restrictions but are available upon request from the corresponding authors.
